# Sustained high demand for intensive care unit resources for the treatment of burn patients during the COVID-19 pandemic in Northern Germany: A single-centre cross-sectional study

**DOI:** 10.1016/j.jpra.2022.07.002

**Published:** 2022-07-10

**Authors:** F. Bucher, K. Dastagir, D. Obed, T. Dieck, P.M. Vogt

**Affiliations:** Department of Plastic, Aesthetic, Hand and Reconstructive Surgery, Hannover Medical School, Carl-Neuberg-Str. 1, D-30625 Hannover, Germany

**Keywords:** Burn, Intensive care, COVID-19, Pandemic

## Abstract

**Introduction:**

The ongoing COVID-19 pandemic caused by SARS-CoV-2 has changed everyday life worldwide. To reduce disease transmission, governments introduced various policies such as social distancing, stay-at-home orders, and travel restrictions. The goal of this study was to investigate the characteristics of burn patients admitted to the burn intensive care unit before and during the COVID-19 pandemic.

**Patients and methods:**

A retrospective descriptive analysis of the hospital's burn registry was performed from 1 March 2019 until 1 January 2022.

**Results:**

A total of 326 patients were included in this study. Eighty-eight patients presented before and 238 patients during the COVID-19 pandemic. The majority of burns occurred during private incidents (80% [2022], 92% [2020]), and burns were most frequently caused by flames (24% [2022], 32.99% [2021]). Work-related injuries occurred less frequently (7.76% [2020], 20% [2022]). Constant results were obtained regarding severity and total body surface area affected (1–80%).

**Conclusion:**

This study highlights high numbers of burn patients admitted to the burn intensive care unit before and during the COVID-19 pandemic. Consequently, burn intensive care units must retain their special position within the national health system and should not be included in resource relocation during the prioritisation of intensive care resources. Multicentre studies should focus on the national impact of COVID-19 on the treatment of burn patients.

## Introduction

The ongoing COVID-19 pandemic caused by SARS-CoV-2 has significantly changed everyday life worldwide since it was first reported in January 2020.^1^ The World Health Organisation (WHO) reported 470 million cases and 6 million deaths in April 2022.^2^ To reduce disease transmission, governments worldwide issued policies such as social distancing, lockdown of cities, stay-at-home orders, and travel restrictions.^3^ The cause, characteristics, and clinical outcome of burn injuries depend on the socioeconomic status of the region, the healthcare system, and the patient's lifestyle.^4^ The COVID-19 pandemic showed various impacts depending on the geographic location.^5^ The German healthcare system has the highest number of intensive care unit beds per citizen, even though a decline is evident. Currently, due to a shortage of intensive care nurses, doctors, and isolation measures in more than 47% of German facilities, two or more beds are not available currently.^6^ In the current situation of shortage of intensive care beds, new triage concepts were applied to provide a justifiable prioritisation concept of intensive care capacities.^7^ Being a small yet highly specialised department, the staff from burn intensive care units is redistributed to COVID-19 isolation wards or within the facility itself. The goal of this study was to identify the influence of the COVID-19 pandemic on burn patients within a highly specialised centre in Northern Germany by performing a retrospective register analysis. The number of patients and their characteristics of burn injuries were compared before and during the pandemic to highlight the importance of burn intensive care units, especially in the presence of a shortage of medical personnel during the COVID-19 pandemic.

## Material and methods

### Study design

A retrospective descriptive analysis of the hospital's burn registry was performed from the 1 March 2019 until the 1 January 2022. The register is maintained by the Department of Plastic, Aesthetic, Hand and Reconstructive Surgery of Hannover Medical School.

Data of all patients who were admitted and treated in the burn intensive care unit of the department were collected anonymously using an online data collection portal. The required data were entered by the attending physician. An informed consent form was signed by the patients or their legal representative. As this was a retrospective analysis of an anonymous database, approval by the local ethics committee was not required.

### Study population

Current burn guidelines require a referral to a specialised burn unit in case of partial-thickness burns > 10% total body surface area (TBSA); full-thickness burns; burn injury of the hand, face, or genitalia; electrical injury; chemical burns; inhalation injury; or burn injury of paediatric patients.^8^ However, emergency doctors may decide individually at the scene based on other factors such as comorbidities if patients require referral to a burn intensive care unit. Emergency doctors may contact the burn centre individually or may use the nationwide call centre run by the Hamburg Fire Department.^9^ Patients included in this study were presented to the burn department of our facility by ambulance or helicopter as a primary referral or secondary transfer.

### Data collection and definitions

Data were collected from the hospital's internal database anonymously, including age at presentation, gender, date of trauma, height, weight, mechanism of burn injury, comorbidities, TBSA affected, presence of inhalation trauma, Abbreviated Burn Severity Index (ABSI), primary or secondary referral, and traumatological injuries. The primary assessment followed by the diagnosis of the burn injury, including calculation of the affected surface and burn depth, was performed by the attending physician and by a board-certified burn surgeon upon arrival. To detect further deepening of the burn wounds, daily re-evaluation was performed. Cases with unknown or missing data were excluded.

### Statistical analysis

Statistical analysis was conducted using GraphPad Prism (GraphPad Software, Inc., La Jolla, California, USA), Microsoft Excel (Microsoft, Redmon, Washington, USA), and Numbers (Apple, Cupertino, California, USA). Descriptive statistics are presented as numbers (percentage) and medians (interquartile range). The number of patients and their characteristics were compared using a two-way analysis of variance (ANOVA). Further, Tukey's and Šídák's multiple comparisons tests were applied. A p-value < 0.05 was considered statistically significant.

## Results

**Fig. 1:**
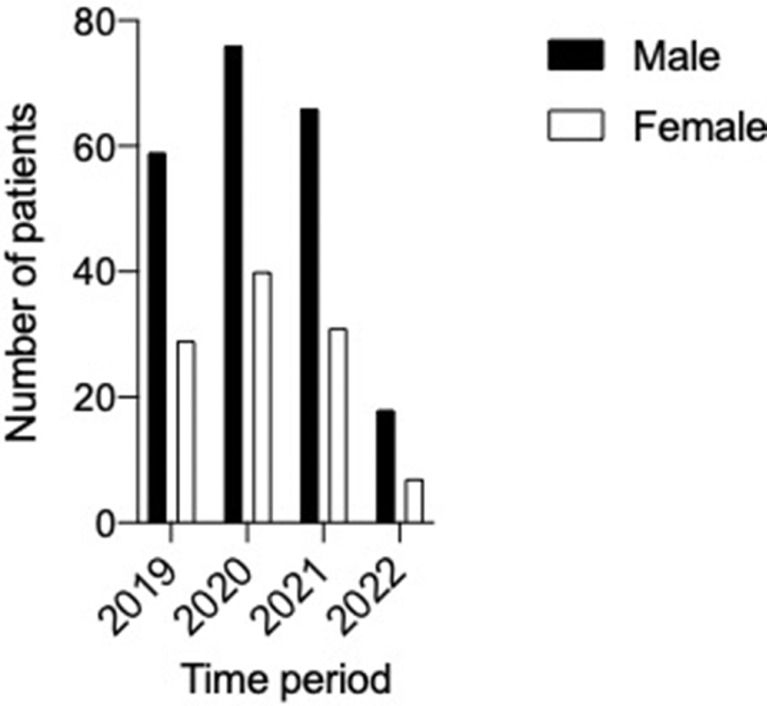
Please provide complete caption

A total of 326 patients were included in this study. Eighty-eight patients presented before the COVID-19 pandemic, whereas 238 patients suffered burn injuries during the pandemic Fig. 1

**Fig. 2:**
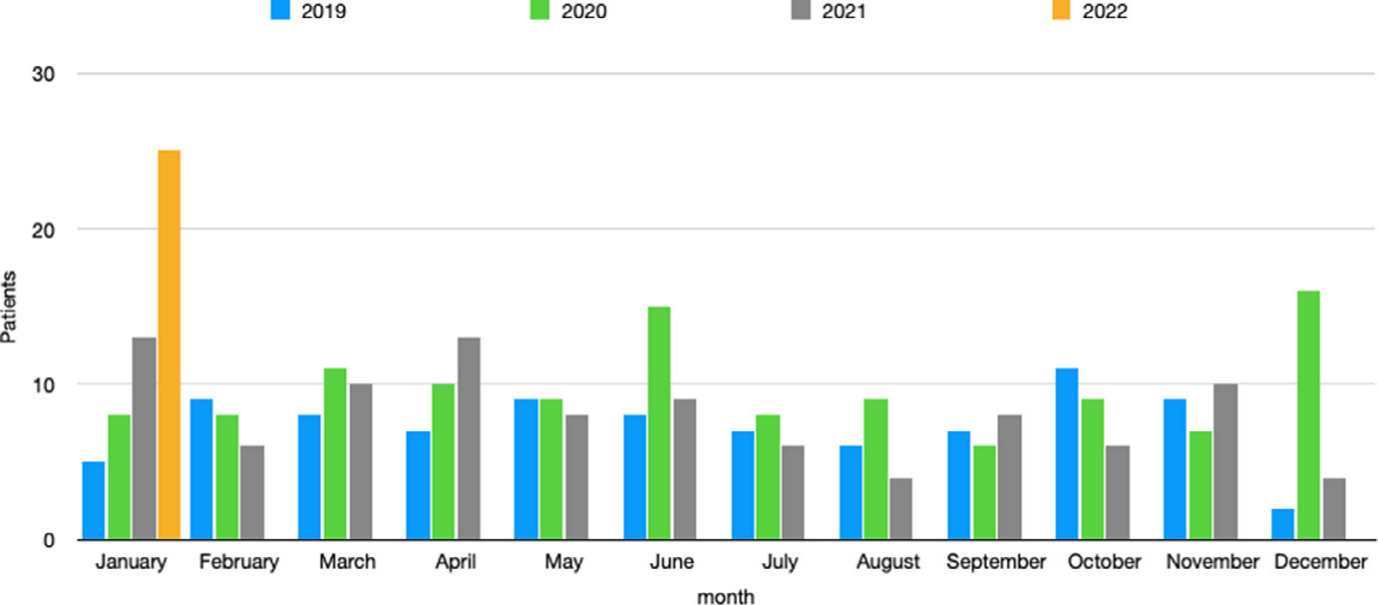
Number of patients per month before and during the COVID-19 pandemic

Fig. 2 summarises the patients treated per month before and during the COVID-19 pandemic. A relative constant patient flow was noticed, with a constant decrease during the summer months. The monthly correlation of burn patients did not show statistically significant correlations (Fig. 2)

**Fig. 3:**
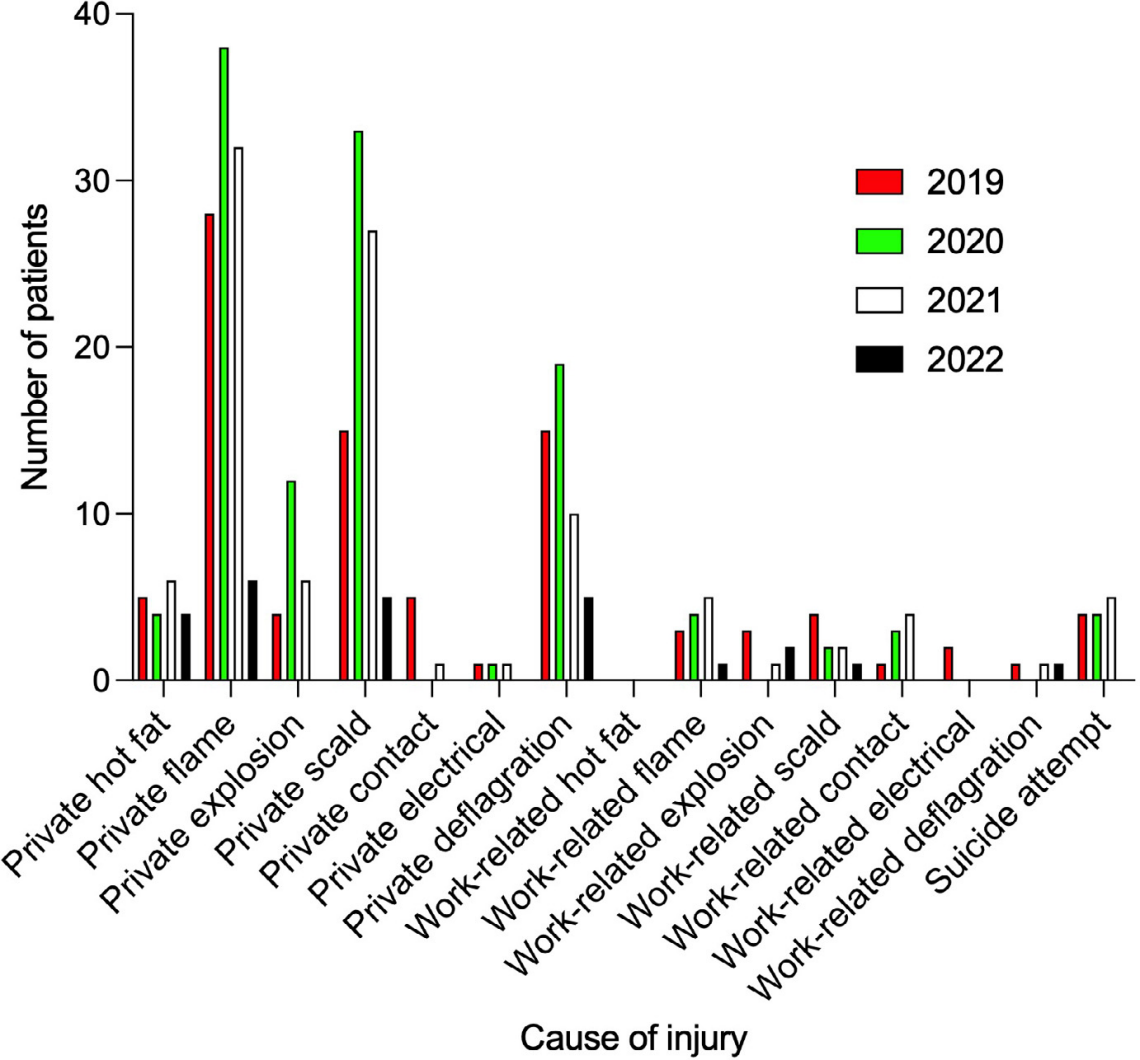
Causes of private and work-related burn injury

**Fig. 4:**
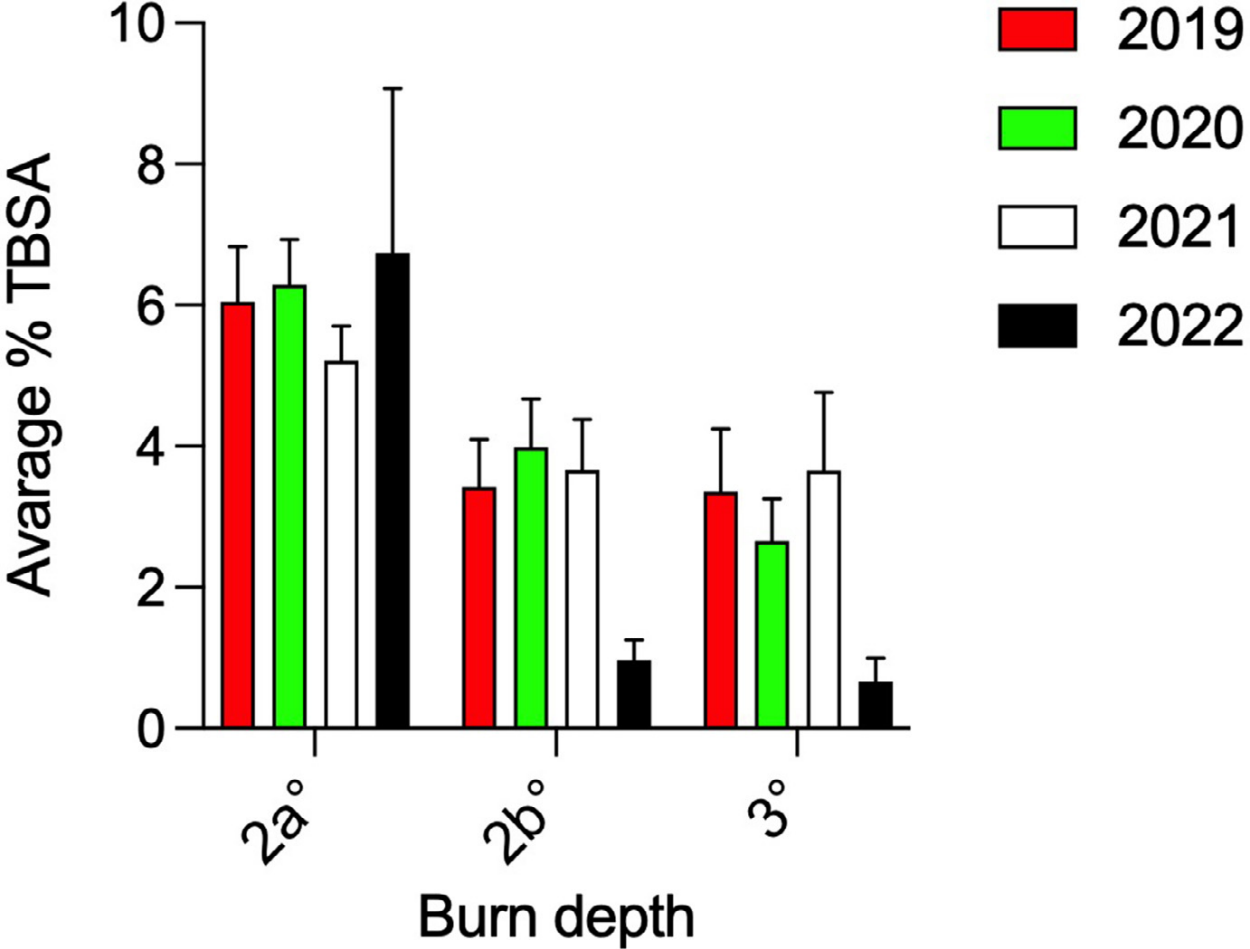
Total burn surface area and burn depth

Table 1 summarises the characteristics of patients treated before and during the COVID-19 pandemic. Overall, 88 patients were treated before and 238 patients during the pandemic. There was no statistically significant correlation between the sex distributions over the years (p-value 0.939–0.998). The patients’ ages ranged from 2 to 94 years and did not show statistically significant correlations over the investigated period of time (p-value 0.581–0.987). The majority of burn injuries occurred during private incidents (80–92%), and burns were most frequently caused by flames (24–32.99%). Work-related injuries occurred less frequently over the investigated period (7.76–20%). Before the pandemic, scald injuries were most frequent (4.55%), whereas during 2020 and 2021, the most frequent work-related injuries were caused by flame exposure (3.45–5.15%). However, in 2022, explosions were most frequently responsible for work-related burn trauma (8%) (Fig. 3). Regarding the severity and TBSA, relatively constant results were reported from 2019 to 2021, ranging from 12.5% to 12.9% (1–80%), whereas for 2022, the mean TBSA was 8.3% (1–25%) (Fig. 4). Overall, burn depth did not show statistically significant correlations (p-value 0.372–0.996). The ratio of primary and secondary hospital transfers was relatively balanced over the years, ranging from 55.67% to 71.59% for primary transfer and from 28.4% to 44.33% for secondary transfer (Table 1), (Fig. 3).

**Table 1:** Characteristics of burn patients before and during the COVID-19 pandemic.

	Before pandemic	During pandemic		
year	2019	2020	2021	2022
cases	88	116	97	25
sex (male)	59	76	66	18
sex (female)	29	40	31	7
Age, years, mean (range)	48,1 (16-90)	47,9 (2-91)	55,5 (3-91)	46,4 (19-94)
Private injury	73 (82,95%)	107 (92,24%)	83 (85,57%)	20 (80%)
hot fat	5 (5,68%)	4 (3,45%)	6 (6,19%)	4 (16%)
flame	28 (31,82%)	38 (32,86%)	32 (32,99%)	6 (24%)
explosion	4 (4,55%)	12 (10,34%)	6 (6,91%)	0
scald	15 (17,05%)	33 (28,45%)	27 (27,84%)	5 (20%)
contact	5 (5,68%)	0	1 (1,03%)	0
electrical	1 (1,14%)	1 (0,86%)	1 (1,03%)	0
deflagration	15 (17,05%)	19 (16,38%9	10 (10,31%)	5 (20%)
Suicide attempt	4 (4,55%)	4 (3,45%)	5 (5,15%)	0
Work-related injury	15 ( (17,05%)	9 (7,76%)	14 (14,43%)	5 (20%)
hot fat	0	0	0	0
flame	3 (3,41%)	4 (3,45%)	5 (5,15%)	1 (4%)
explosion	3 (3,41%)	0	1 (1,03%)	2 (8%)
scald	4 (4,55%)	2 (1,72%)	2 (2,06%)	1 (4%)
contact	1 (1,14%)	3 (2,59%)	4 (4,12%)	0
electrical	2 (2,28%)	0	0	0
deflagration	1 (1,14%)	0	1 (1,03%)	1 (4%)
Partial thickness burn 2a°	6.0%	6.2%	5.2%	6.7%
Partial thickness burn 2b°	3.4%	3.9%	3.6%	1.0%
Full thickness burn 3°	3.4%	2.7%	3.7%	0.7%
TBSA (%)	12,9 (1-62)	12,9 (1-80)	12,5 (1-77)	8,3 (1-25)
ABSI Score	4,9 (1-12)	4,7 (1-12)	5,4 (1-14)	5 (3-9)
primary transfer	63 (71,59%)	68 (58,62%)	54 (55,67%)	16 (64%)
secondary transfer	25 (28,4%)	48 (41,38%)	43 (44,33%)	9 (36%)

**Fig. 5:**
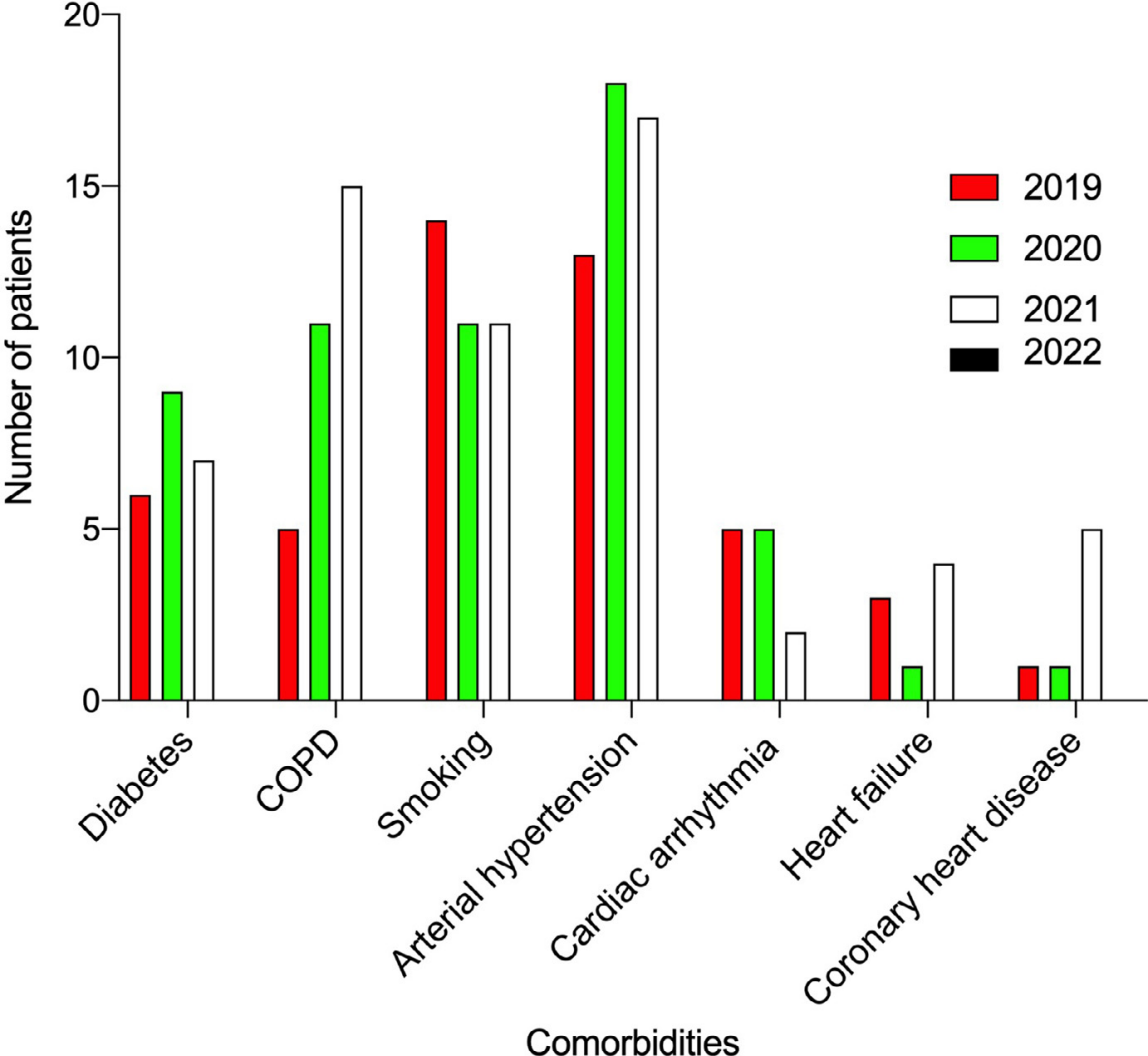
Associated comorbidities accompanying the burn injury

The most common comorbidities of the admitted patients are summarised in Fig. 5. Even though patients often presented with multiple comorbidities, no statistically significant correlation was identified (p-values 0.322–0.971). Two patients presented with burn injury and concomitant SARS-CoV-2 infection (0.8%) (Fig. 4, Fig. 5).

## Discussion

This descriptive study investigated the detailed characteristics of burn patients admitted to a specialised centre in Northern Germany before and during the COVID-19 pandemic. Despite the efforts made by the government issuing policies, such as social distancing, city lockdowns, and stay-at-home orders, a continuously high number of burn patients admitted to the burn intensive care unit was observed.

Similar studies were published by other burn centres from economically developed countries such as the UK, the USA, Canada, and Japan.^5^^,^^10^, ^11^, ^12^, ^13^ D'Asta et al. reported a decrease in adult burn patients by 60% during the lockdown, although the number of paediatric burn cases increased by 8% at the same time.^11^ Our specialised centre does not treat paediatric burn patients, and thus this study does not give insight regarding the incidence of paediatric burn patients. A slight decrease of admitted burn patients by 5.8% was reported by Codner et al.^12^ Further, a regional burn centre located in the UK reported a 30% decrease in adult burns during the COVID-19 pandemic.^13^ On the other hand, a study from a US centre reported more frequent paediatric burns during the COVID-19 pandemic.^14^ Reduction of severity and incidence was also observed for trauma patients during COVID-19 lockdown.^15^

The major cause of private burn injuries was reported as flame burns, which is most likely explained by the prolonged stay of people in closed spaces in their homes. Similar results were reported in the literature.^16^ During the COVID-19 pandemic, a new mechanism of burn sustained by the usage of hand sanitiser followed by thermal exposition by cooking or lighting of a cigarette was reported, as patients were not aware of the flammable composition of hand sanitiser.^17^ However, in our study population, this new mechanism was not found to cause burn injuries.

Due to lockdown policies, work-related burn injuries were expected to decrease; however, our study did not identify statistically significant results. This might be explained by the fact that the lockdown policies mainly affected the retail, culture, and food sectors and spared the heavy industry where hygiene concepts or short-time working was introduced early in Germany. No relationship could be identified in previously published studies, which may be explained by the generally lower incidence of work-related burn injuries.^5^^,^^18^ Further, we expected a rise in burn patients with concomitant SARS-CoV-2 infection. Interestingly, only two patients presented with burn injury and concomitant SARS-CoV-2 infection (0.8%).

The first wave of the COVID-19 pandemic put the global medical system to an especially hard test. Being confronted with a large number of critically ill patients, restructuring clinical pathways and services was necessary in order to maintain high standards of patient care. New consulting approaches via telemedicine follow-up proved to be a reliable tool for increasing patient-led self-care, reducing hospital admissions, and minimising clinical attendance of medical personnel.^19^ Since the beginning of the COVID-19 pandemic, burn intensive care units were forced to introduce protocols for the admission of new patients in order to detect positive cases early and prevent spread among patients and healthcare personnel. Establishing and respecting these protocols consumes important intensive care resources.^20^ When confronted with an extreme shortage of intensive care resources, multiple concepts of prioritisation and triage were discussed in Germany to improve aggregate outcomes.^21^ Fortunately, in Germany, prioritisation is performed only locally, meaning that resources such as intensive care staff and beds are transferred within the hospital in order to manage the patient flow efficiently. Unfortunately, the COVID-19 pandemic shifted the focus away from the critical burn patients who are yet severely affected in all vital functions, which can range from altered metabolism, hypothermia, and impaired pulmonary function to hemodynamic instability. SARS-CoV-2 infection combined with a severe burn injury is associated with high mortality, even if admitted early to an intensive care unit.^20^

Our study highlighted continuously high numbers of burn patients admitted to the burn intensive care unit before and during the COVID-19 pandemic. Burn injuries represent a small yet complex patient collective that requires highly specialised intensive care staff and infrastructure as an intensive care unit. Consequently, the burn intensive care unit must maintain a special position within the national health system. Decision-makers must be discouraged from moving medical personnel from burn intensive care units to other specialised units since the incidence of burn patients was not affected for this patient cohort. As soon as the SARS-CoV-2 infection rate declines, resources should be reallocated to burn intensive care units in order to provide the highest standard of care for critically ill burn patients.

This study has several limitations. It was retrospective study with a relatively small sample. This study also represents a single-centre analysis; however, it is the only specialised centre in the whole county, making the data still representative of a large geographic area. Our centre was confronted with a severe shortage of rehabilitation clinics, making secondary patients transfer the bottleneck for new patient admission; hence, the sample size is potentially biased and might be considered larger.

## Conclusions

The ongoing COVID-19 pandemic has dramatically changed everyday life around the world.

Despite the efforts made by the government issuing policies, such as social distancing, city lockdowns, and stay-at-home orders, a continuously high number of burn patients admitted to the burn intensive care unit before and during the COVID-19 pandemic was observed.

Flame burns caused the majority of burn injuries, most likely due to the prolonged stay of people in their homes. Further, work-related burn injuries remained constant as lockdown policies mainly affected retail, culture, and food sector while sparing the heavy industry. The expected rise in burn patients with concomitant Sars-CoV-2 infection did not set in, as only two burn patients were tested positive for COVID-19.

The results of this study underline the special position of burn intensive care units within the national health system, being small, yet highly specialised departments. The COVID-19 pandemic has shifted the decision-makers focus away from the critical burn patients, making it necessary to highlight the importance of the highly specialised intensive care units.

These units should not be included in prioritisation measures, and for providing the highest standard of care, resources should be relocated.

## Funding

There was no funding source for this study.

## Institutional review board statement

Being a retrospective database analysis with anonymous patient data, no approval by the Ethics Committee was required according to the Institutional Review Board.

## Informed consent statement

Written informed consent was obtained from all subjects involved in this study.

## Data availability statement

Data were collected from the hospital's internal database anonymously for each patient. After informed consent is obtained, the attending physicians will enter the above-mentioned data into a database. It can then be extracted anonymously for research purposes.

## CRediT authorship contribution statement

**F. Bucher:** Conceptualization, Funding acquisition, Formal analysis, Writing – original draft. **K. Dastagir:** Software, Formal analysis, Visualization, Data curation. **D. Obed:** Methodology, Writing – review & editing. **T. Dieck:** Supervision, Data curation, Validation, Writing – review & editing. **P.M. Vogt:** Conceptualization, Supervision, Writing – review & editing, Project administration.

## Declaration of Competing Interest

The author and co-authors declare that they have no competing financial interests or personal relationships that could have influenced the results reported in this manuscript.
